# Tristetraprolin Mediates Radiation-Induced TNF-α Production in Lung Macrophages

**DOI:** 10.1371/journal.pone.0057290

**Published:** 2013-02-28

**Authors:** Dipankar Ray, Shirish Shukla, Uday Sankar Allam, Abigail Helman, Susmita Gurjar Ramanand, Linda Tran, Michael Bassetti, Pranathi Meda Krishnamurthy, Matthew Rumschlag, Michelle Paulsen, Lei Sun, Thomas P. Shanley, Mats Ljungman, Mukesh K. Nyati, Ming Zhang, Theodore S. Lawrence

**Affiliations:** 1 Department of Radiation Oncology, University of Michigan, Ann Arbor, Michigan, United States of America; 2 Department of Pediatrics and Communicable Diseases, University of Michigan, Ann Arbor, Michigan, United States of America; National Cancer Institute, United States of America

## Abstract

The efficacy of radiation therapy for lung cancer is limited by radiation-induced lung toxicity (RILT). Although tumor necrosis factor-alpha (TNF-α) signaling plays a critical role in RILT, the molecular regulators of radiation-induced TNF-α production remain unknown. We investigated the role of a major TNF-α regulator, Tristetraprolin (TTP), in radiation-induced TNF-α production by macrophages. For *in vitro* studies we irradiated (4 Gy) either a mouse lung macrophage cell line, MH-S or macrophages isolated from TTP knockout mice, and studied the effects of radiation on TTP and TNF-α levels. To study the *in vivo* relevance, mouse lungs were irradiated with a single dose (15 Gy) and assessed at varying times for TTP alterations. Irradiation of MH-S cells caused TTP to undergo an inhibitory phosphorylation at Ser-178 and proteasome-mediated degradation, which resulted in increased TNF-α mRNA stabilization and secretion. Similarly, MH-S cells treated with TTP siRNA or macrophages isolated from *ttp* (−/−) mice had higher basal levels of TNF-α, which was increased minimally after irradiation. Conversely, cells overexpressing TTP mutants defective in undergoing phosphorylation released significantly lower levels of TNF-α. Inhibition of p38, a known kinase for TTP, by either siRNA or a small molecule inhibitor abrogated radiation-induced TNF-α release by MH-S cells. Lung irradiation induced TTP^Ser178^ phosphorylation and protein degradation and a simultaneous increase in TNF-α production in C57BL/6 mice starting 24 h post-radiation. In conclusion, irradiation of lung macrophages causes TTP inactivation via p38-mediated phosphorylation and proteasome-mediated degradation, leading to TNF-α production. These findings suggest that agents capable of blocking TTP phosphorylation or stabilizing TTP after irradiation could decrease RILT.

## Introduction

Radiation-induced lung toxicity (RILT) limits the use of potentially curative high-dose (chemo)-radiation for patients with thoracic malignancies [1] and occurs in up to 30% of treated patients [2], [3]. Radiation causes the production of key cytokines, including TGF-ß1 and TNF-α, which appear to be major contributors to RILT [4]–[7]. Increased production, activation and/or signaling of TGF-ß1 plays a central role in RILT, and increased levels of TGF-ß1 in the plasma of irradiated lung cancer patients predict subsequent lung toxicity [8], [9]. TNF-α is a known regulator of various inflammatory diseases including arthritis, psoriasis, inflammatory bowel disease [10]–[12], and is also involved in various pulmonary inflammatory diseases including bronchitis, chronic obstructive pulmonary disease (COPD), asthma and acute lung injury (ALI) [13]. Furthermore, inhibition of TNF-α receptor either by genetic manipulation (knockout) or via antisense oligonucleotide (ASO) treatment can protect the lung from radiation toxicity [14].

Although little is known regarding the mechanism by which radiation increases TNF-α production, there has been substantial investigation into TNF-α regulation in other systems. Stimulation of lung macrophages, the major TNF-α producing cells in mice [15], [16], with lipopolysaccharide (LPS) leads to enhanced production of TNF-α, due to the post-transcriptional regulation of TNF-α transcript [17]. Tristetraprolin (TTP), a zinc finger containing RNA-binding protein, plays a critical role in degrading the TNF-α transcript in this system [18]. TTP binds directly to the AU-rich element in the 3′-UTR of TNF-α transcript leading to its destabilization and rapid degradation [19]. TTP knockout mice have high endogenous levels of TNF-α, due directly to the absence of TTP-mediated inhibition of TNF-α production [18]. In contrast to most proteins, phosphorylation of TTP leads to its inactivation (therefore producing effects similar to TTP knockdown). This is because, compared to the phosphorylated TTP, the unphosphorylated or de-phosphorylated form of TTP recruits more efficiently the deadenylase and mRNA decapping complexes to the AU-rich element containing TNF-α transcript to cause rapid degradation [20], [21]. Thus, absent or phosphorylated TTP is associated with increased TNF-α production, whereas unphosphorylated or de-phosphorylated TTP decreases TNF-α levels.

The most important regulator of TTP phosphorylation in LPS-treated lung macrophages appears to be the p38-MK2 pathway [22]–[24]. The p38-MK2 pathway is reported to phosphorylate TTP at two important serine residues (Ser^52^ and Ser^178^), and mutation of these serine residues to alanine enhances the effectiveness of TTP in inhibiting LPS-mediated TNF-α production [25], [26]. Furthermore, p38-mediated TTP phosphorylation controls TTP localization and stability [27].

Because of the importance of TTP in the regulation of LPS-induced TNF-α production in macrophages, we thought it was possible that radiation might stimulate TNF-α production through similar mechanisms. Therefore, we assessed the changes in expression and p38-mediated phosphorylation of TTP in response to radiation in a mouse macrophage cell line MH-S. We also assessed the effects of radiation on macrophages isolated from *ttp* knockout mice and further analyzed TTP expression and phosphorylation in mouse lung, in an attempt to evaluate the importance of post-translational modification-dependent TTP inactivation as a mechanism of radiation-induced TNF-α production.

## Materials and Methods

### Ethics Statement

Here we confirm that all the animal studies conducted here were approved by the University Committee on Use and Care of Animals (UCUCA) of the University of Michigan (protocol # PRO00001915).

### Cells and Reagents

The mouse alveolar macrophage cell line MH-S was obtained from ATCC and cultured in regular DMEM supplemented with 10% FBS (Sigma). Chinese hamster ovary (CHO) cells were cultured in RPMI medium with 10% FBS. Rabbit polyclonal TTP antibodies were purchased from Santa Cruz Biotechnology (sc-14030), Abcam Inc. (ab36558 and ab83579) and Sigma (T5452). Rabbit polyclonal Phospho-TTP^Ser178^ antibody was a kind gift from Dr. Georg Stoecklin (Germany). Total, phospho-specific p38 antibodies and antibodies against GAPDH were purchased from Cell Signaling Technology (Danvers, MA). Rat monoclonal (Cl: A3-1) F4/80 antibody was purchased from Abcam Inc. (ab6640). siRNA against mouse TTP (sc-36761), mouse p38 (sc-29434) and control siRNA (sc-37007) were purchased from Santa Cruz Biotechnology. Kinase inhibitors were purchased from the following sources: SB203580 (Cat. V1161, Promega Corp.), Wortmannin (Cat. 9951, Cell Signaling), GSK3 inhibitors, SB216763 and SB415286 (Cat. 1616 and 1617, Tocris Chem.). ^35^S-methionine (7 mCi/0.680 ml) was purchased from MP Biomedicals.

### Constructs

Constructs containing Myc/His-tagged wild type (pcDNA3-TTP-wt-Myc/His) and serine-to-alanine mutants of TTP (S52A, S178A and S52/178A [AA]) have been described [25]. PCMV-hTTP (wild type) construct was purchased from Origene Inc.

### BrU Pulse Labeling to Determine Rate of TNF-α mRNA Synthesis

To determine the rate of TNF-α mRNA synthesis and simultaneously determine the stability of transcribed RNA, a BrU pulse-chase labeling assay was followed as described previously [28]. Briefly, MH-S cells were either sham irradiated or irradiated with 4 Gy radiation and 48 h post-radiation cells were incubated with 2 mM BrU in conditioned medium for 30 min to label nascent RNA. Cells were then washed 3 times in PBS and either collected directly (0 hr time point) or chased in conditioned medium containing 20 mM uridine for 6 hr at 37°C. Total RNA was isolated using TRIzol reagent, and the BrU-containing RNA was isolated using magnetic beads (Dynabeads, Goat anti-mouse IgG, Invitrogen) conjugated to anti-BrdU monoclonal antibody (BD Biosciences). Conversion of the isolated BrU-containing mRNA into cDNA and real-time PCR analyses were performed as described below.

### Measurement of mRNA Levels of TNF-α in Irradiated MHS Cells

Total RNA was isolated using the RNAeasy Mini kit (Qiagen, Valencia, CA) according to the manufacturer’s instructions. RNA (1 µg) was reverse transcribed to cDNA using the High Capacity cDNA Archive kit (Applied Biosystems, Foster City, CA) and purified (Millipore Centrifugal Filter Units, Billerica, MA). Diluted cDNA was used to amplify GAPDH (GAPDH F: 5′ CTG GAG AAA CCT GCC AA GTA 3′ and GAPDH R: 5′ TGT TGC TGT AGC CGT ATT CA 3′), TTP (TTP F: 5′AAA TTC AGT GTT TGG GTG GA 3′ and TTP R: TGT AAC CCC AGA ACT TGG AA 3′) and TNF-α (set I: TNF F: 5′ CCC ACT CTG ACC CCT TTA CT 3′ and TNF R: 5′ TTT GAG TCC TTG ATG GTG GT 3′; set II: F: GGT CCC CAA AGG GAT GAG AAG TTC 3′ and R: 5′CCA CTT GGT GGT TTG CTA CGA CG 3′) by quantitative reverse-transcription PCR (qRT-PCR) using SYBR green chemistry (Applied Biosystems). The PCR products were resolved by electrophoresis on 1.5% agarose gels, and melting curve analysis was carried out to confirm the specificity of the product. The δδCt method was used to analyze the data as described elsewhere [29], and GAPDH was used as control.

### siRNA Transfection

MH-S cells were transfected using Lipofectamine RNA_i_max (Invitrogen) with 20 nM of either a control siRNA duplex (sc-37007) or an siRNA duplex targeting mouse TTP (sc-36761) or mouse p38 (sc-29434) using protocol as described previously. 24 h following a second transfection, cells were either sham radiated or irradiated with 4 Gy, and the culture supernatant, RNA or cell lysates were harvested as described previously [30].

### Bone Marrow Derived Macrophages (BMDM) Isolation

Murine bone marrow derived macrophages were prepared from bone marrow of 8–10 weeks old mice following the procedure described previously [31], [32]. Briefly, tibias and femurs were collected aseptically from the *ttp* wild type (+/+) and null (−/−) mice. The bone marrow was flushed out and the cells were cultured in RPMI supplemented with 30% L929 cell conditioned medium, 20% heat inactivated FBS in 100 mm culture dishes for 7–8 days. A day before radiation, equal number of cells was replated and was either sham-irradiated or irradiated with 4 Gy. To confirm macrophage differentiation, few cells were immunostained with F4/80 marker. RNA samples were collected at different time points and quantitated for TNF-α transcripts as described above.

### Mouse Lung Radiation and Tissue Collection

C57BL/6 male mice 6 to 8 weeks of age were purchased from Charles River laboratories and were either sham irradiated or irradiated (Philips RT250 orthovoltage unit that produces 250 kV X-rays, Kimtron Medical) with a single dose of 15 Gy to the whole lung as described previously [30]. For tissue collection, the right ventricle of the heart was perfused with PBS to clear blood from the lungs, and tissue lysates were prepared using RIPA buffer (25 mM Tris-HCl, pH 7.5, 150 mM NaCl, 1% NP-40, 1% sodium deoxycholate, 0.1% SDS).

### TNF-α ELISA

TNF-α secreted into the culture supernatant was quantified using an ELISA kit (R&D Systems) according to manufacturer’s instructions.

### Protein Analyses

For immunoblotting, cell lysates were prepared as described previously [33]. In brief, culture dishes were placed on ice and washed once with ice cold PBS. Required amounts of lysis buffer were added to each plate, and cells were harvested by scraping. Cells were lysed using a cup-type sonicator followed by clearing of debris by centrifugation at 4°C. Protein amount was quantified using Bradford reagents according to manufacturer’s instructions.

### ^35^S-methionine Pulse-chase Assay

For metabolic labeling, 48 h after 4 Gy radiation, MH-S cells were washed twice with PBS and twice with DMEM [methionine (met)/cysteine (cys) free] supplemented with 10% dialyzed serum. Cells were incubated with 3 ml of such medium for 1 h to deplete the intracellular pools of sulfur-containing amino acids. ^35^S-met (0.2 mCi) was added into each plate, and cells were incubated for 3 h. Labeling medium was then removed and the cells were washed with ice cold PBS, and cell lysates were prepared as described above. Cell lysates were then subjected to immunoprecipitation using TNF-α antibody. The immunocomplexes were resolved in 12% bis-tris gel, and the gel was fixed and incubated with Amplify (GE Healthcare, Cat. # NAMP100). The gel was then vacuum dried at 80°C and developed by autoradiography.

For pulse-chase experiment to calculate the TTP protein half life in sham-irradiated or 4 Gy irradiated MH-S cells, 1×10^6^ cells were plated in DMEM medium containing 10% FBS and incubated overnight. Cells were then washed twice with PBS and twice with DMEM (-met/−cys) with 10% dialyzed serum. Cells were incubated for 1 h with 3 ml of the Met/Cys-free DMEM containing dialyzed serum and pulse labeled with 0.2 mCi of ^35^S-met for 1 h. A set of plates were then sham-irradiated and another set were received 4 Gy of radiation. Labeling medium was then removed and cells were washed twice with PBS and twice with DMEM medium with 10% FBS and 100 mM cold methionine. Cells were then incubated with excess Met-containing DMEM medium with 10% FBS for different time periods as indicated. For the 0 h time point, cell lysates were prepared immediately after radiation both from sham-irradiated and irradiated plates. Immunoprecipitation was performed using anti-TTP antibody, followed by SDS-PAGE analysis and autoradiography was performed as described above.

### Immunostaining

Immunostaining was performed as described previously [34]. Briefly, slides containing cryo-preserved mouse lung sections were incubated at room temperature for 5 min, then fixed with ice cold methanol for 20 min at −20°C and were washed in PBS, blocked for 1 h, and incubated in primary antibody at 4°C overnight. The slides were then washed again in PBS, incubated with the secondary antibody for 1 h, rewashed, and prepared with a coverslip after a drop of ProLong Gold anti-fade reagent with 4′, 6-diamidino-2-phenylindole (Molecular Probes) was added to each sample. Fluorescence images were acquired using a DS-Fi1 (Nikon, Melville, NY) camera fitted on an Olympus 1X-71 microscope.

### Statistical Analysis

Data are expressed as means ± S.D. The unpaired Student’s t-test was used to compare the differences between two groups, and a *P* value of <0.05 was considered as significant.

## Results

### Radiation Induces TNF-α mRNA and Protein Levels in MH-S Cells

**Figure 1:**
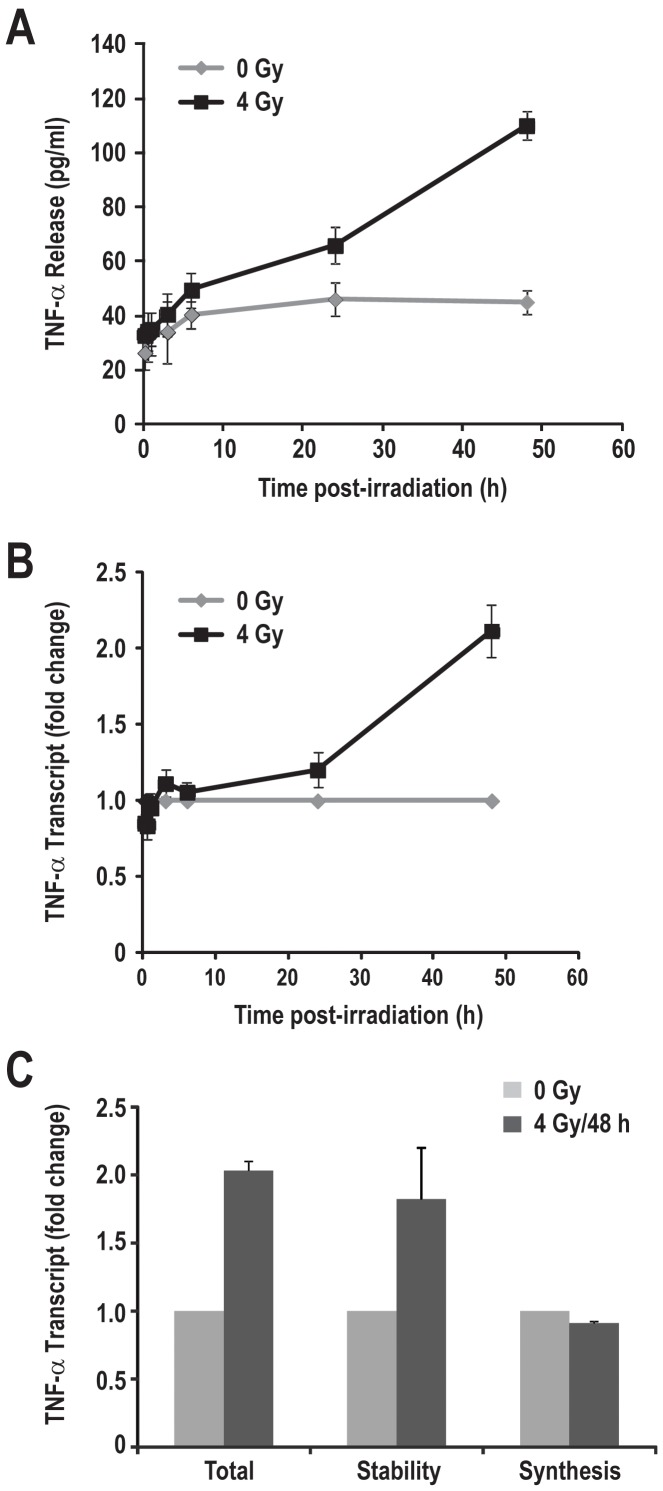
Radiation increased the TNF-α transcript and its release by MH-S cells 48 h post–radiation. (A) MH-S cells were left untreated (0 Gy) or irradiated (4 Gy) and culture supernatants were collected at the indicated time points. Released TNF-α levels were quantified using ELISA kits according to the manufacturer’s instruction. (B) Cells were treated as above, and RNA was isolated and quantified at the indicated time points as described in the materials and methods. (C) MH-S cells were either sham irradiated or irradiated with 4 Gy. 48 h post-irradiation TNF-α mRNA level, stability and synthesis was determined using BrU pulse-chase labeling experiment as described previously [28].

As lung macrophages are the major TNF-α producing cell upon radiation, we began by assessing the effect of radiation on TNF-α secretion by the mouse lung macrophage cell line, MH-S. We treated MH-S with different doses (2 and 4 Gy) of radiation and quantified TNF-α secretion into the culture supernatant using ELISA between 30 min till 48 h post-radiation. Two Gy had no major effects on TNF-α release until 48 h after radiation (data not shown); however 4 Gy consistently induced the secretion of the cytokine at 48 h post-irradiation (Fig. 1). To understand the time course of radiation-induced TNF-α secretion, we harvested the culture supernatants at various time points post-radiation (Fig. 1). We found that there was a 2.4 fold (control, 45±4.5 pg/ml; +RT, 110±5.2 pg/ml) increase in TNF-α secretion (Fig. 1A) and a 2.1±0.18 fold increase in TNF-α transcript (Fig. 1B) 48 h after radiation. To distinguish the involvements of mRNA synthesis versus alteration of mRNA stability, we performed BrU pulse-chase labeling assay (see methods). We found that radiation increased the stability of TNF-α transcript to 1.82±0.37 fold (n = 3), whereas no significant change in synthesis was noted at 48 h post-irradiation (Fig. 1C). Metabolic labeling of MH-S cells with ^35^S-methionine followed by immunoprecipitation of TNF-α using specific antibody showed that radiation did not change the rate of TNF-α translation up to 48 h post-irradiation (Fig. S1). From these analyses we concluded that irradiation of MH-S cells increases TNF-α transcript stability, which may be responsible for the increased secretion of the cytokine.

### Tristetraprolin Regulates Radiation-induced TNF-α Production by MH-S Cells

**Figure 2:**
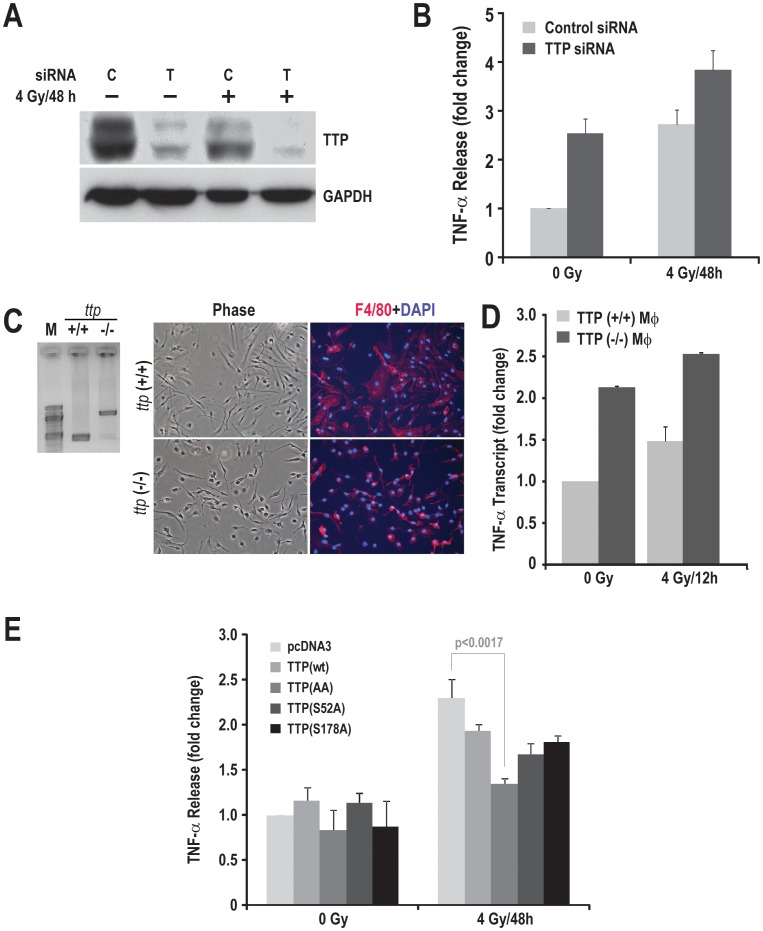
Tristetraprolin negatively regulates radiation-induced TNF-α production. (A) MH-S cells were treated with either control (C) or TTP (T) siRNA and 24 h post-transfection cells were left un-irradiated or irradiated with 4 Gy. Cell lysates were prepared 48 h post-radiation and immunoblotted with the indicated antibodies. (B) TNF-α levels secreted in the culture supernatant were quantitated on the above mentioned samples and represented as fold change considering the sham irradiated control siRNA-treated sample as 1. (C) Bone marrow from *ttp* (+/+) and *ttp* (−/−) mice were isolated and differentiated into macrophages as described in the materials and methods. Left panel shows the PCR based genotyping of the mice used in the study and the right panel confirms macrophage differentiation using F4/80 immunofluorescence staining. (D) Bone marrow derived macrophages were either sham irradiated or radiated with 4 Gy. 12 h post-irradiation RNA samples were isolated and TNF-α transcript levels were quantitated as described in materials and methods. (E) MH-S cells were transfected with either vector control DNA or with various TTP constructs (WT, Ser52Ala, Ser178Ala, and Ser52/178Ala). 24 h post-transfection, cells were exposed to 4 Gy, and TNF-α secretion was quantified in culture supernatant 48 h post-radiation.

Tristetraprolin (TTP) regulates mRNA stability of various inflammatory cytokines, most notably TNF-α (see Introduction). As the irradiation-induced TNF-α increase in MH-S cells was associated with increased TNF-α mRNA stability (Fig. 1C), we wished to characterize the involvement of TTP in radiation-induced TNF-α production. We used three major strategies: (i) siRNA-mediated gene silencing (ii) macrophages isolated from TTP-deficient mice and (iii) over-expression of super-active TTP mutants. TTP siRNA was able to down-regulate about 70% of the endogenous protein levels in MH-S cells (Fig. 2A, lane 2) which caused approximately a 2.5 fold increase in TNF-α secretion by macrophages even without radiation. Furthermore, in the control siRNA-treated group, 4 Gy caused a decrease of about 50% of endogenous TTP levels compared to the sham-irradiated group (Fig. 2A, lane 3), which was associated with a 2.5 fold increase in TNF-α secretion. This change was similar to that observed after TTP siRNA treatment without radiation. Interestingly, only about 5% of TTP remained after the combination of radiation and TTP siRNA (Fig. 2A, lane 4), which was associated with an additional 1.6 fold increase in TNF-α secretion (*P*<0.001; Fig. 2B). To better understand the role of TTP, we isolated bone marrow from *ttp* (+/+) and *ttp* (−/−) mice and differentiated them to macrophages as evidenced by the F4/80 positive staining (Fig. 2C). As shown in Fig. 2D, macrophages isolated from wild type mice showed a trend of increased TNF-α mRNA levels (1.48±0.17) upon 4 Gy of irradiation similar to irradiated MH-S cells. As reported earlier, the basal TNF-α transcript level was higher (2.13±0.01) in *ttp*-null macrophages, which upon radiation increased to 2.53±0.02 fold 12 h post-irradiation (Fig. 2D). Conversely, transient over-expression of either wild type (wt) TTP or mutants of TTP that are unable to be phosphorylated [Ser^52^A (S^52^A), Ser^178^A (S^178^A), and SS^52/178^AA] and inactivated caused a significant inhibition of radiation-induced TNF-α secretion 48 h post-radiation compared to vector transfected cells (Fig. 2E). Vector control transfected MH-S cells showed a 2.2±0.3 fold increase in TNF-α secretion upon radiation as compared to non-irradiated cells; cells transfected with TTP-SS^52/178^AA showed only a modest increase of about 1.3±0.1 fold (*P*<0.0017). These experiments demonstrate TTP involvement in radiation-induced TNF-α secretion.

### Radiation Causes Increased TTP Phosphorylation and Proteasome-mediated Protein Down-regulation

**Figure 3:**
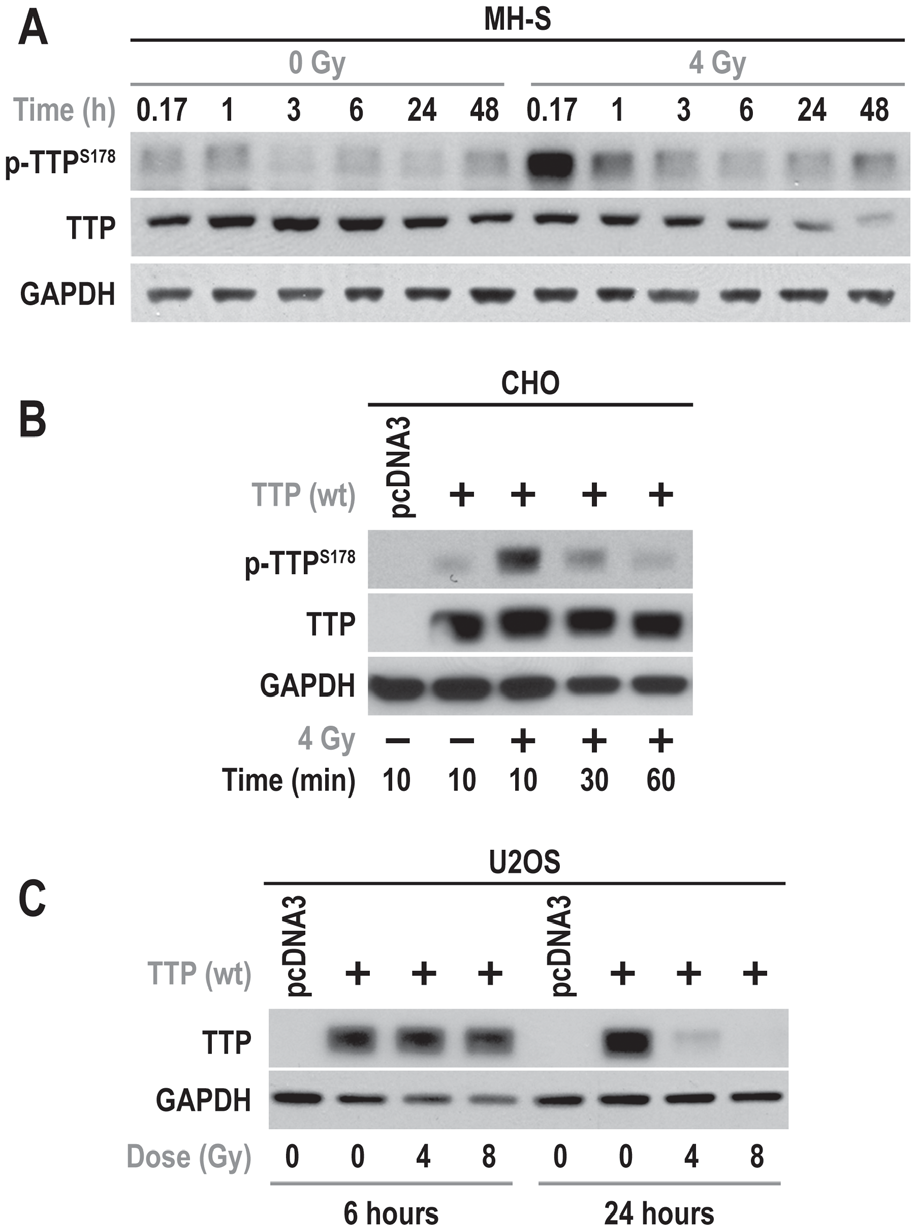
Radiation caused increased phosphorylation and down-regulation of TTP in MH-S cells. (A) MH-S cells were either sham irradiated or treated with 4 Gy. Cells were harvested at indicated time points and immunoblotted using indicated antibodies. (B) CHO cells overexpressing mouse TTP were treated with 4 Gy, harvested at different time points, and immunoblotted using indicated antibodies. (C) U2OS cells overexpressing human TTP were irradiated with either 4 or 8 Gy and immunoblotted using indicated antibodies.

We have used two systems (endogenous and over-expressed) to study the effect of radiation on post-translational modifications of TTP. To determine if the effect of radiation was mediated by TTP phosphorylation, cell lysates were prepared from both non-irradiated and irradiated MH-S cells at various times starting at 10 min after radiation. Within 10 minutes after radiation, there was a significant increase in Ser^178^ phosphorylation of TTP, which gradually declined with time (Fig. 3A). A similar trend in TTP phosphorylation kinetics was detected when TTP over-expressing CHO cells (which express undetectable levels of TTP) were irradiated (Fig. 3B). Interestingly, at later time points (between 24–48 h post-radiation), we found a significant down-regulation of TTP protein levels in MH-S cells (Fig. 3A). There was a similar dose-dependent radiation-induced down-regulation of overexpressed TTP in U2OS cells (an osteosarcoma cell line having undetectable endogenous TTP) at 24 h after 4 and 8 Gy (Fig. 3C). These observations show that radiation causes an early enhancement of TTP Ser^178^ phosphorylation and late total TTP down-regulation.

**Figure 4:**
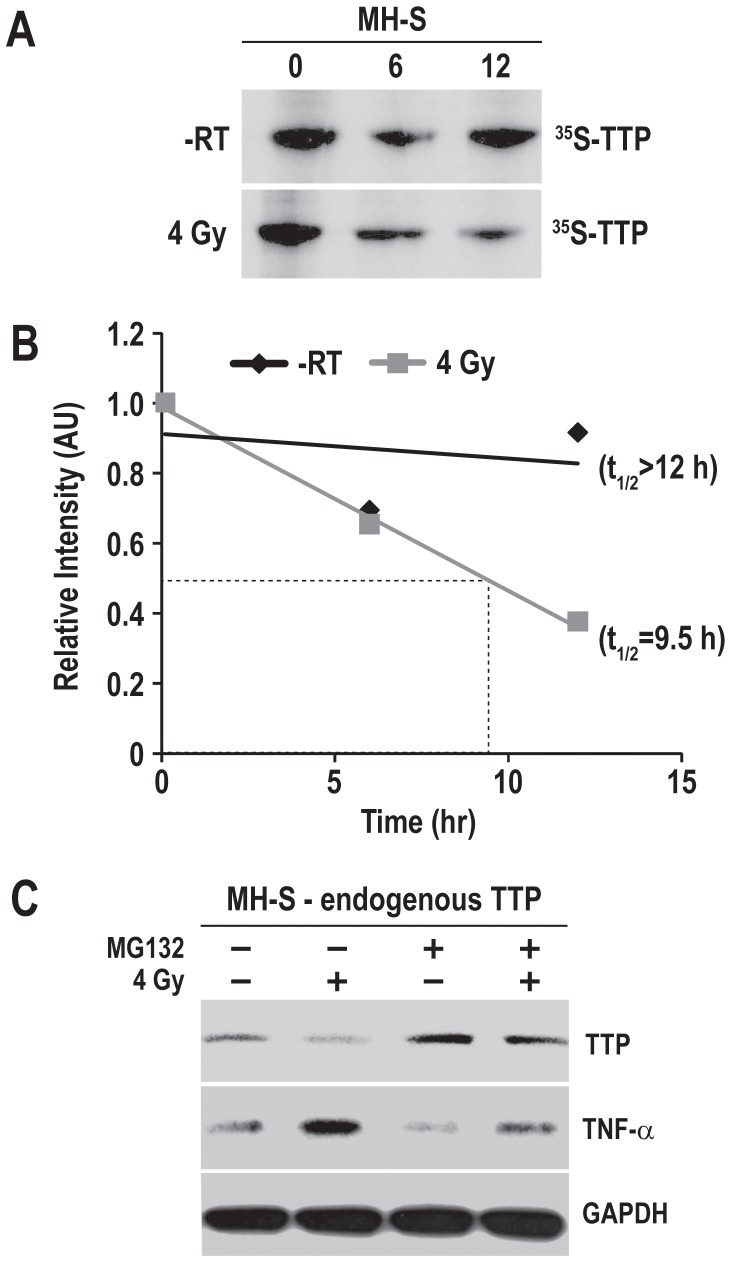
Radiation-induced TTP degradation and TNF-α secretion is inhibited significantly by the proteasome inhibitor MG132. (A) MH-S cells were metabolically labeled with ^35^S-methionine, were either sham-irradiated or irradiated with 4 Gy, and chased with cold methionine for indicated time periods. After the chase period cell lysates were subjected to immunoprecipitation using TTP antibody, immunocomplexes were resolved by SDS-PAGE and autoradiography. (B) TTP protein’s half life in sham-irradiated (-RT) and irradiated (4 Gy) groups were determined by densitometric scanning of the autoradiographs followed by quantitation using Image J1.32j software (NIH, Bethesda, MD). Relative protein levels were determined in comparison to sample isolated immediately after the pulse labeling (0 h chase). (C) MH-S cells were either sham-irradiated or radiated with 4 Gy, and 44 h after radiation 2 µM of MG132 was added. Cell lysates were collected 4 h after MG132 addition and immunoblotted for TTP and TNF-α. GAPDH was used as loading control.

To better understand the radiation-induced TTP down-regulation, endogenous TTP protein’s half-life in MH-S cells was determined using ^35^S-methionine pulse-chase assay. As shown in Fig. 4A and calculated in Fig. 4B, in sham-irradiated MH-S cells, TTP protein’s half life was calculated to be >12 h, whereas 4 Gy reduced the half-life to about 9.5 h.To determine if proteasomal degradation was responsible for radiation-induced TTP down-regulation, we treated MH-S cells with MG132 (a proteasomal inhibitor) for 4 h prior to harvest. MG132 protected against radiation-induced TTP down-regulation and reduced radiation-induced TNF-α release (Fig. 4C). These data suggest that radiation reduces the TTP protein’s half-life via proteasomal degradation and further confirm the role of TTP in suppressing TNF-α release.

### p38 Kinase Controls Radiation-induced TTP Phosphorylation and TNF-α Secretion by MH-S Cells

**Figure 5:**
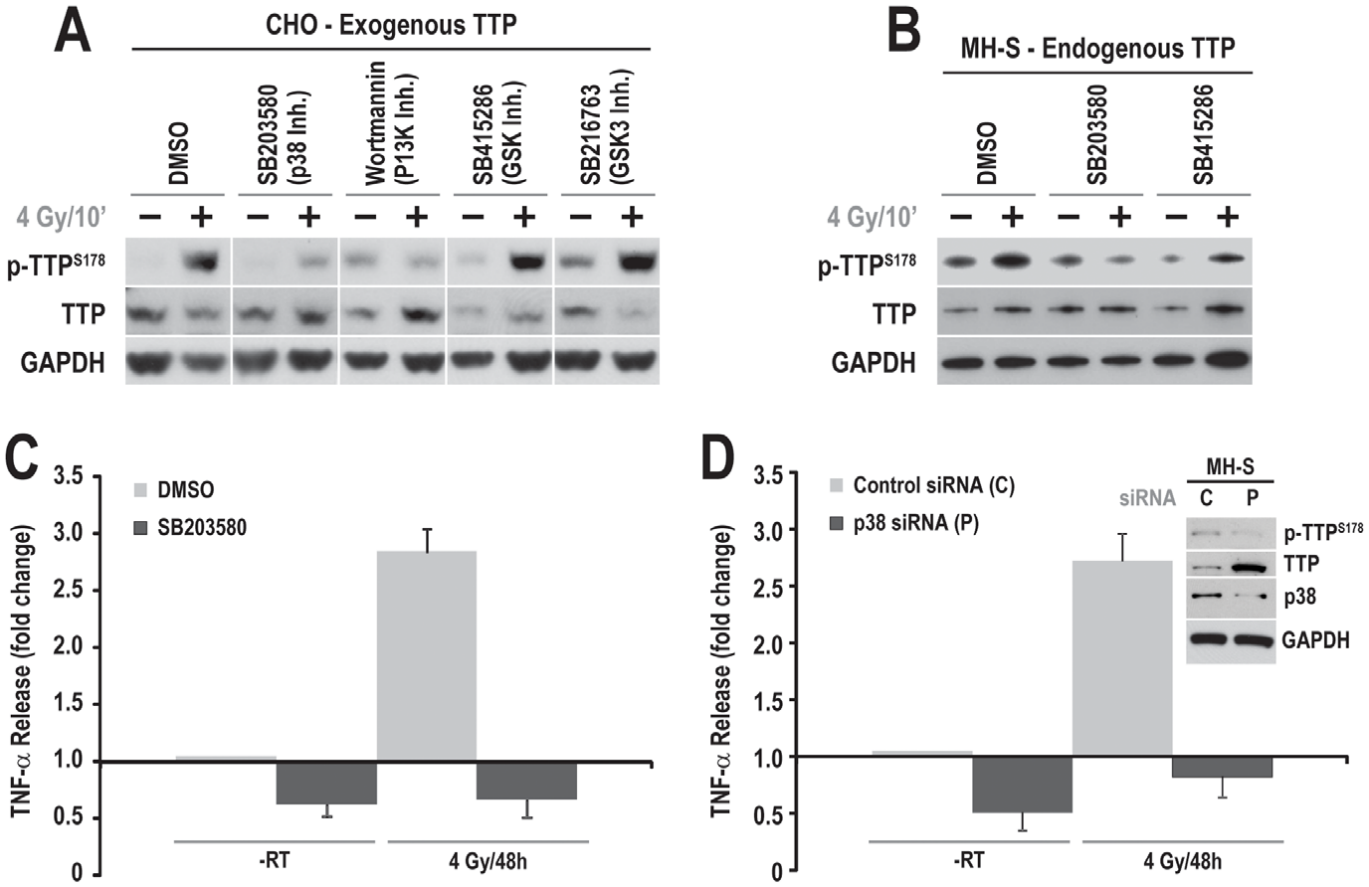
p38 kinase controls radiation-induced TTP phosphorylation and TNF-α secretion by MH-S cells. (A) CHO cells overexpressing TTP were treated with 4 Gy in the presence of either DMSO (vehicle control) or p38 inhibitor (SB203580), or PI3K inhibitor (Wortmannin), or GSK3ß inhibitors (SB415286, SB216763). Cell lysates were prepared 10 min post-radiation and immunoblotted using indicated antibodies. (B) MH-S cells were pretreated with either p38 or GSK3ß inhibitors as above and cell lysates were prepared 10 min post-radiation and immunoblotted with the indicated antibodies. (C) MH-S cells were irradiated with 4 Gy in the presence or absence of a p38 inhibitor (SB203580), and radiation-induced TNF-α secretion was quantified using ELISA. (D) MH-S cells were treated with either control (C) or TTP (T) siRNA. 24 h post-transfection, cells were either left un-irradiated or radiated with 4 Gy. Culture supernatants were collected 48 h post-radiation, and TNF-α levels were quantified. In the inset, the effectiveness of p38 siRNA is shown in cell lysates isolated from C or T siRNA treated cells.

After having shown that radiation causes TNF-α secretion upon TTP phosphorylation, we wanted to identify the kinase responsible for radiation-induced TTP phosphorylation. To this end, we pre-treated CHO cells overexpressing TTP with various kinase inhibitors (e.g. p38 inhibitor, SB203580; PI3K inhibitor, Wortmannin and GSK3 inhibitors, SB214667 and SB51223) to determine which, if any, inhibit radiation-induced TTP phosphorylation. Among these kinase inhibitors, SB203580 and Wortmannin significantly blocked the radiation-induced Ser^178^-phosphorylation of TTP, whereas GSK3 inhibitors had no effect (Fig. 5A). Similar results were obtained with endogenous TTP phosphorylation when MH-S cells were pre-treated with the inhibitors (Fig. 5B). As we expected, SB203580 also inhibited radiation-induced TNF-α secretion by MH-S cells (Fig. 5C). To further characterize the involvement of p38 in radiation-induced TTP phosphorylation, we used siRNA to down-regulate p38. We confirmed that knockdown of p38 by siRNA (Fig. 5D inset) can inhibit radiation-induced TNF-α secretion by MH-S cells. A significant increase in TTP level was detected in p38 siRNA treated cells (Fig. 5D inset). These findings demonstrate that p38 is responsible for radiation-induced TTP phosphorylation, which causes its inactivation allowing increased TNF-α mRNA levels and secretion.

### Radiation Leads to p38 Phosphorylation, TTP Phosphorylation and Down Regulation, and TNF-α Secretion in Mouse Lung

**Figure 6:**
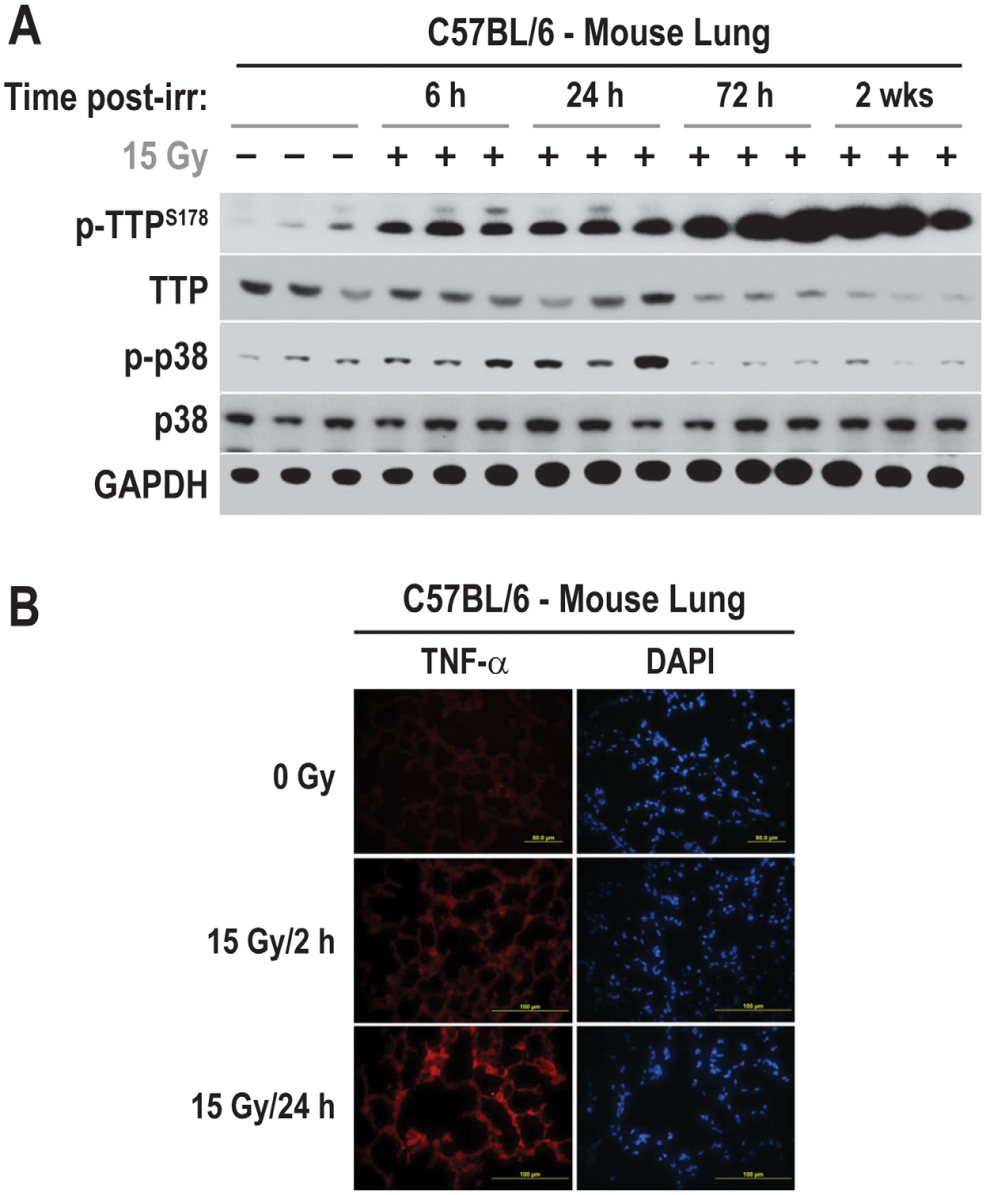
Radiation causes significant down-regulation of TTP in irradiated mouse lung. (A) C57BL/6 mice were either sham-irradiated or the whole lung was irradiated with a single dose of 15 Gy. Tissue lysates were prepared from three individual animals at each indicated time points post-radiation and immunoblotted using indicated antibodies. (B) Mouse lung cryo-sections from sham-radiated or 15 Gy single fraction radiated mice were isolated at indicated time points and immune-staining were performed as described in materials and methods.

To determine if radiation induces p38 phosphorylation, TTP phosphorylation and TNF-α down-regulation *in vivo*, mice were irradiated, and the lungs were assessed at different time points post-radiation. As shown in Fig. 6A, there was a significant up-regulation of TTP (Ser^178^) phosphorylation and a down-regulation of steady state TTP levels between 72 h-2 weeks post-radiation. Furthermore, reprobing the membrane with Thr^180^ and Tyr^182^ phospho-specific p38 antibody showed increased phosphorylation as well. Finally, immunofluorescence staining of cryo-preserved lung sections confirmed that radiation caused an increase in TNF-α (Fig. 6B). These observations demonstrate that p38 phosphoryation (associated with activation), TTP phosphorylation and down regulation (associated with inactivation), and TNF-α induction occur *in vivo* as well as *in vitro*.

## Discussion

**Figure 7:**
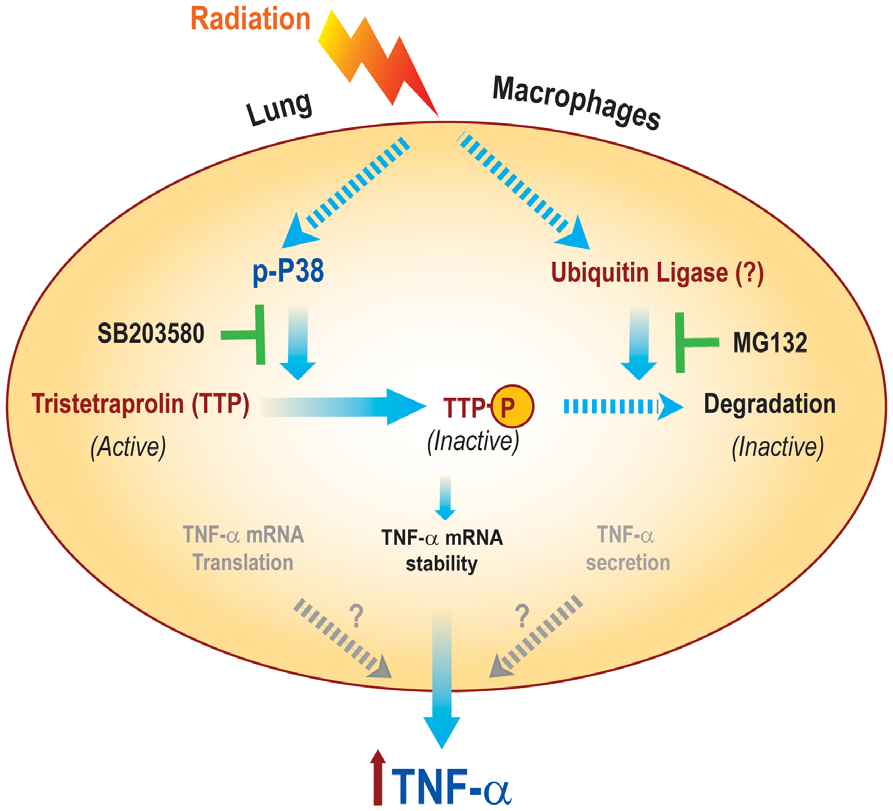
Schematic model explaining the role of post-translational TTP modifications (phosphorylation and degradation) in radiation-induced TTP inactivation as an upstream regulator involved in increased TNF-α secretion by mouse lung macrophages.

Altered production of TNF-α, a pro-inflammatory cytokine has long been associated with various pathological conditions that affect the normal lung function, including radiation pneumonitis and lung fibrosis [35]. We have previously demonstrated that radiation-induced lung injury could be mitigated via inhibition of the TNF-α receptor I (TNFRI) either by genetic ablation of the receptor or by specific antisense oligonucleotide (ASO) treatment [14]. Furthermore, inhibition of TNF-α signaling did not decrease tumor cell killing, thus providing therapeutic selectivity [14]. More importantly, inhibition of TNF-α by Etanercept (Enbrel) improves lung function for patients with idiopathic pulmonary syndrome, which results from lung injury after high dose chemotherapy [36]. Taken together, these studies suggest a pivotal role of TNF-α in lung complications resulting from cancer treatment. In this report we have elucidated the mechanism of radiation-induced TNF-α production described by a schematic model shown in Fig. 7. We propose that radiation activates the p38 pathway, which inactivates Tristetraprolin (TTP), an RNA binding protein that is a major negative regulator of TNF-α mRNA. Inactivation occurs through both an inhibitory phosphorylation at position Ser^178^ and by subsequent proteasome-mediated TTP degradation, releasing TTP’s negative regulation and allowing increased TNF-α mRNA stability and secretion. These observations point not only to a critical role of TTP in RILT, but they also suggest that blocking TTP inactivation may protect the normal lung from radiation injury.

Macrophages are the major TNF-α producing cell in response to lung irradiation [16], [37]. Activated macrophages are also believed to play a critical role in the pathogenesis of other diseases including rheumatoid arthritis, where release of cytokines, particularly TNF-α, instigates immunological reactions that cause destruction of synovial tissue [38]. Similarly, in a rheumatoid arthritis animal model, lipopolysaccharide (LPS) treatment causes similar pathological symptoms via increased TNF-α production [39]. Genetic ablation of TTP in mice causes arthritis that can be rescued by treatment with a TNF-α neutralizing antibody, demonstrating the major role of TNF-α [18]. Because of such similarities, we wanted to characterize the involvement of TTP in radiation-induced TNF-α production in the lung. During the LPS-induced inflammatory response, TTP is reported to undergo inactivation via p38-MK2 mediated phosphorylation at Serine 178 position [23], [27], thus allowing enhanced TNF-α mRNA levels.

Our study shows that radiation and LPS have some similarities but also substantial differences in their effect on TNF-α. For example, both radiation and LPS treatment are capable of inducing the release of TNF-α upon TTP inactivation. However, unlike LPS, the amplitude of radiation-induced increase in the TNF-α secretion is relatively smaller and is not associated with a significant increase at the transcript level. Similarly, we found that although radiation and LPS are capable of inducing p38-mediated inhibitory TTP phosphorylation; however, unlike LPS, which up-regulates TTP levels, radiation causes proteasome-mediated TTP degradation. Ionizing radiation has the ability to cause damage to both DNA and the cell membrane leading to an inflammatory response [40]–[42]. In contrast, LPS-induced inflammatory signaling is mainly initiated via a cell surface receptor, CD14/TLR4 [43]. Whether such differences are responsible for differential ubiquitin ligase activation leading to TTP ubiquitination and degradation only in the case of ionizing radiation remains an open question.

Radiation has long been known to activate various stress-activated signaling via phosphorylation of mitogen-activated protein (MAP) kinases including p38, ERK and JNK [44]. In the case of LPS-mediated enhanced TNF-α production, activation of both p38 and ERK pathways have been reported, which play a significant role in phosphorylating TTP at Serine-178 leading to its inactivation and resulting in stabilization of TNF-α transcript [23], [27], [44]. In our study, although we observed that radiation induced TTP phosphorylation at Serine-178 in MH-S cells, which was significantly abrogated by a p38 inhibitor, the kinetics of TTP phosphorylation and the release of TNF-α were disconnected. TTP^S178^ phosphorylation occurred as early as 10 min post-radiation, whereas TNF-α release was not prominent until 48 h post-radiation. It is, therefore, likely that there are intermediate steps between radiation-induced TTP inactivation and TNF-α release. We speculate that S178 phosphorylation may be a priming event for further TTP phosphorylation at other sites to induce its inactivation, the identity and time kinetics of such phosphorylation so far remain unknown. Interestingly, in samples isolated from irradiated mouse lung tissue, radiation-induced TTP phosphorylation was a late event (72 h-2 weeks post-irradiation), and the kinetics of TTP inactivation coincided well with the previously published literature on an enhanced second burst of a biphasic TNF-α release by mouse lung following a single dose of radiation [37]. Whether such differences in radiation-induced TTP phosphorylation kinetics between the *in vitro* and *in vivo* systems reflect differences in the cellular cross-talk remain to be elucidated. Alternatively, it is also plausible to hypothesize that radiation-induced TTP phosphorylation may not be sufficient enough to completely inactivate TTP function to cause early increase in TNF-α production; instead at later time points TTP is completely inactive via proteasome-mediated degradation thus allowing TNF-α release at later time points.

Although our studies have identified TTP as a major regulator of radiation-induced TNF-α production, it also raised the existence of alternative signaling in the process. We have not only observed increased basal level of TNF-α secretion by TTP-deficient macrophages as reported previously, we have also noted that radiation can further promote modest but consistent increase in TNF-α secretion in TTP-deficient cells (either MH-S treated with Ttp siRNA or bone marrow macrophages from *ttp*-null animals). Such observations point to the fact that although TTP may be a major negative regulator of TNF-α levels, there may be additional regulator(s) involved in radiation-induced TNF-α production. Previous studies have identified that ADAM17/TACE as a metalloprotease involved in TNF-α shedding, thus helps in cytokine secretion [45]. It is plausible that radiation may be regulating expression/activity of such factor(s) to regulate TNF-α secretion in a TTP-independent manner. Additionally, it was demonstrated that p38-mediated inhibitory phosphorylation of TTP blocks its ability to replace another RNA binding protein called human antigen R (HuR) from binding to TNF-α mRNA precursor. Such association not only stabilizes TNF-α transcript, it also promotes efficient translation of TNF-α [46]. Although 4 Gy does not alter the rate of TNF-α translation 48 h post-irradiation (Fig. S1), the possibility exists that radiation may alter the rate of TNF-α translation at early time points, which require further investigation.

So far, the only FDA approved radioprotector is Amifostine (WR-2721), a free radical scavenger [47]. However, this drug does not offer any radioprotection for the lung [48], [49]. TNF-α signaling has been successfully targeted by anti-TNF antibodies (e.g. Etanercept) in the case of rheumatoid arthritis [50], [51]. Our recent findings about the role of TTP in controlling TNF-α level in RILT suggest that preventing radiation-induced TTP inactivation and degradation could be a promising way to develop a radioprotector. The strategy of maintaining TTP’s ability to suppress TNF-α activity has appeal with respect to selectivity. For the normal tissue, TTP inhibits radiation-induced cytokine production and reduces inflammatory responses. At the same time, prevention of TTP inactivation would be anticipated to be deleterious for cancer cells, where TTP is known to negatively regulate the expression of urokinase plasminogen activator (uPA), uPA receptor, matrix metalloproteinase-1 (MMP-1) and VEGF, thus decreasing tumor cell growth, invasion and metastasis [52], [53]. As we have observed that radiation induces TTP inactivation via p38-mediated phosphorylation and proteasomal degradation to produce and release increased TNF-α, we could propose to target p38. Indeed there are several clinical trials using different p38 inhibitors to treat patients with various illnesses, including acute lung toxicity and rheumatoid arthritis, where TNF-α plays a major role [54]. Alternatively, the inhibition of the ubiquitin ligase controlling TTP stability (identity unknown) would also be predicted to prevent radiation-induced TTP inactivation and should suppress TNF-α activity. Either of these strategies could be combined with anti-TNF therapy to block TNF-α signaling effectively, which should provide better radioprotection for the lung. Finally, in this study we have focused our attention on understanding the role of TTP in radiation-induced early lung toxicity, which is characterized by lung pneumonitis. However, fibrosis is an important cause of late radiation-induced lung function failure. It will be important to determine if late fibrosis is a result of early pneumonitis, or if TTP plays a direct role in the fibrotic process.

## Supporting Information

Figure S1 **4 Gy irradiation does not alter TNF-α rate of translation in MH-S cells.** MH-S cells were either sham-irradiated or irradiated with 4 Gy and left for 48 h. Cells were then metabolically labeled with ^35^S-Met for 3 h. Cell lysates were then subjected to immunoprecipitation using TNF-α antibody, separated in SDS-PAGE and autoradiographed.
(TIF)
