# Supplementary figures and images for: Tristetraprolin Mediates Radiation-Induced TNF-α Production in Lung Macrophages
Source: PLoS One. 2013 Feb 28;8(2):e57290. doi: 10.1371/journal.pone.0057290 (PMC3585360; doi:10.1371/journal.pone.0057290)

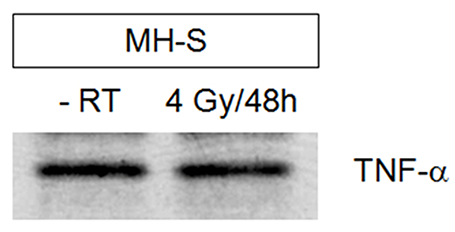

Supplement: Figure S1 — 4 Gy irradiation does not alter TNF-α rate of translation in MH-S cells. MH-S cells were either sham-irradiated or irradiated with 4 Gy and left for 48 h. Cells were then metabolically labeled with 35S-Met for 3 h. Cell lysates were then subjected to immunoprecipitation using TNF-α antibody, separated in SDS-PAGE and autoradiographed. (TIF) [file pone.0057290.s001.tif]
